# Instantaneous Remedy for Lumbago

**Published:** 1889-09

**Authors:** 


**Instantaneous Remedy for Lumbago.**—Collodion, tincture of iodine, liquid ammonia, equal parts. To be applied widely over the parts with a camel's hair brush.—*Ex.*

